# Performance of Bioactive, Fluoride-Releasing, and Conventional Pit and Fissure Sealants: Effect of Enamel Conditioning via phosphoric acid and Er, Cr: YSGG laser in Primary Teeth

**DOI:** 10.12669/pjms.42.6.15798

**Published:** 2026-06

**Authors:** Manal Al-Mutairi

**Affiliations:** Manal Al-Mutairi, Pediatric Dentistry and Orthodontics Department, College of Dentistry, King Saud University, Saudi Arabia

**Keywords:** Microleakage, Bond strength, Pit and fissure sealants, Fluoride, Bioactive sealants

## Abstract

**Objective:**

To investigate the influence of two enamel surface conditioning techniques, i.e., 37% phosphoric acid (PA) etching and Er, Cr: YSGG laser (ECL) conditioning, on microleakage and micro tensile bond strength (μTBS) of conventional, fluoride-releasing, and bioactive pit and fissure sealants (PFS) applied to primary molars enamel.

**Methodology:**

The study lasted two months, from 1^st^ December 2025 to 1^st^ February 2026. A total of ninety caries-free primary mandibular molars with preshedding mobility and indicated for extraction were selected. Teeth exhibiting developmental enamel defects, occlusal caries, existing restorations, or signs of attrition or erosion were excluded from the study. The teeth were arbitrarily assigned to two groups according to the enamel conditioning protocol (n=45): Group-1: PA and Group-2: ECL. Each experimental group was subdivided into three subgroups according to the PFS used (n=15). Subgroup A: Conventional PFS, Subgroup B: Fluoride-releasing PFS, and Subgroup C: Bioactive sealant. Microleakage was evaluated in five samples from each subgroup. Sixty sealed molars were longitudinally sectioned for micro tensile bond strength testing. Statistical analysis was performed using *ANOVA* followed by *Tukey’s post hoc* test for multiple comparisons, p<0.05.

**Results:**

The highest marginal leakage was recorded in Group-2A (ECL + Conventional PFS) (30.54 ± 0.42), with the lowest bond strength (7.01 ± 0.75 MPa). In contrast, the lowest marginal leakage was observed in Group 1C (PA + Bioactive PFS), with the highest bond strength (11.52 ± 0.43 MPa).

**Conclusion:**

Fluoride-releasing and bioactive pit and fissure sealants demonstrated significantly superior performance compared to conventional sealants. Additionally, enamel pretreatment with either phosphoric acid or an Er:Cr:YSGG laser yielded comparable bond strength and marginal leakage.

## INTRODUCTION

Dental caries remains the most prevalent chronic disease worldwide, with a complex multifactorial etiology involving biological, environmental, and behavioral determinants.^1^ Pit and fissure surfaces represent anatomical sites of heightened susceptibility, accounting for 56–70% of carious lesions among children aged 5–17 years, despite the well-documented preventive efficacy of dental sealants.^2^ The structural composition of primary tooth enamel differs substantially from that of permanent dentition, characterized by reduced thickness, decreased mineralization, elevated organic content, increased permeability, and a wider aprismatic surface layer.^3^ These inherent microstructural variations directly influence acid-etching patterns, adhesive bonding mechanisms, and ultimately the retention performance of pit and fissure sealants (PFS).^4^ Consequently, optimizing enamel surface conditioning protocols constitutes a critical determinant of clinical success in pediatric preventive dentistry.

Conventional enamel pretreatment employs 37% phosphoric acid (PA) etching to generate surface microporosities that facilitate micromechanical retention. While effective, phosphoric acid conditioning increases enamel permeability, rendering surfaces vulnerable to demineralization if incomplete resin infiltration occurs.^5^ Additionally, the technique demands meticulous moisture control and optimal isolation, presenting practical challenges in pediatrics.^6^ These limitations have prompted investigation of alternative conditioning modalities, including Er, Cr: YSGG laser (ECL) irradiation operating at 2780 nm wavelength.^7^ ECL generates micro-retentive, debris-free enamel surfaces through hydrokinetic ablation mechanisms without chemical exposure. Although reported bond strengths are comparable to or slightly lower than those with acid etching, parameter optimization remains crucial—studies demonstrate that 3.5 W for 15 seconds enhances adhesion in primary teeth. In contrast, suboptimal settings (e.g., 2.5 W) compromise microtensile bond strength (μTBS).^8^,^9^

Contemporary PFS have evolved beyond conventional third-generation light-polymerized resin-based materials, which function as passive mechanical barriers without inherent anticariogenic properties. Fourth-generation fluoride-releasing sealants exhibit limited sustained fluoride release, providing minimal long-term therapeutic benefit.^2^,^10^ Recently developed bioactive sealants represent advanced formulations engineered to release calcium and phosphate ions, thereby maintaining mineral equilibrium between tooth structure and oral environment.^11^,^12^ Despite promising in vitro bond-strength data following artificial aging, clinical investigations report inferior retention rates for bioactive materials compared with conventional alternatives, yielding contradictory evidence regarding their clinical performance.

This investigation tested dual null hypotheses: (1) ECL conditioning produces microleakage, and μTBS similar to PA etching within identical sealant groups; and (2) fluoride-releasing, bioactive, and conventional sealants demonstrate comparable bond strength and microleakage within identical conditioning protocols (ECL and PA). Therefore, this study aimed to evaluate how different enamel surface conditioning techniques affect the marginal seal integrity and tensile strength of various pit-and-fissure sealant formulations.

## METHODOLOGY

This in-vitro investigation adhered to the CRIS (Checklist for Reporting In-vitro Studies) guidelines. The current in vitro study protocol received approval from the King Saud University Institutional Review Board (IRB # R26-IRB-140; Dated September 15, 2025). The study lasted two months, from 1^st^ December 2025 to 1^st^ February 2026.

### Ethical Approval:

The current in vitro study protocol received approval from the King Saud University Institutional Review Board (IRB # R26-IRB-140).

### Sample Selection and Preparation:

Ninety caries-free primary mandibular molars exhibiting physiological preshedding mobility and scheduled for extraction were collected. Inclusion criteria encompassed teeth with intact occlusal surfaces free from developmental enamel defects, carious lesions, existing restorations, or pathological wear patterns (attrition/erosion). Following extraction, specimens underwent ultrasonic scaling to eliminate adherent soft tissues and biofilm, followed by prophylactic polishing with fluoride-free pumice slurry. Teeth were stored in freshly prepared 0.5% chloramine-T trihydrate solution for one week to prevent bacterial proliferation. Before the experimental procedures, occlusal surfaces were prophylaxed, and pits and fissures were mechanically debrided with a dental explorer and rinsed with an air-water spray. Specimens were embedded in self-curing acrylic resin (Demotec 20, Demotec Dental, Nidderau, Germany) within standardized cylindrical polyvinyl molds, with the roots positioned at the cementoenamel junction and the occlusal planes oriented perpendicular to the longitudinal axis. To ensure procedural consistency and minimize operator fatigue, a single calibrated operator sealed up to 10 specimens daily. Specimens were randomly allocated to two experimental groups using computer-generated randomization, following the enamel conditioning protocol (n=45 per group).

### Enamel Surface Conditioning Protocols:

### Group-1 (Phosphoric Acid Etching):

Occlusal enamel surfaces received a 37% phosphoric acid gel (Actino Gel, Prevest DenPro, Jammu, India) application for 20 seconds, followed by a 10-second water rinse and a 5-second air-dry using a triple syringe until a characteristic dull, chalky-white etched appearance was achieved.

### Group-2 (Er, Cr: YSGG Laser Conditioning):

Erbium, chromium-doped yttrium-scandium-gallium-garnet laser irradiation (Waterlase MD, Biolase, Irvine, CA, USA; wavelength: 2780 nm) was performed using G6 sapphire tips (600 µm diameter) with the following parameters: power output 3.5 W, pulse duration 140 µs, energy per pulse 50–75 mJ, repetition rate 20 Hz. Air-water spray cooling was maintained at 50–60% water and 40–50% air to prevent thermal damage. Laser energy delivery was performed in a non-contact mode, with the laser positioned 1 mm perpendicular to the occlusal surfaces, using a continuous scanning motion for 15 seconds. Post-irradiation, specimens were rinsed thoroughly and gently air-dried.^13^

### Pit and Fissure Sealant Application:

Each conditioning group was subdivided according to sealant type (n=15 per subgroup):

### Subgroup-A (Conventional Sealant):

Light-cured resin-based sealant (Helioseal, Ivoclar Vivadent, Schaan, Liechtenstein) was applied using a fine sable-hair brush technique, ensuring complete pit-and-fissure penetration with slight extension onto cusp inclines. Photopolymerization was achieved using an LED curing unit (Rogin Dental Shenzhen, China) for 20 seconds at manufacturer-specified intensity.

### Subgroup-B (Fluoride-Releasing Sealant):

Fluoride-releasing resin sealant (Clinpro Sealant, 3M ESPE, St. Paul, MN, USA) was applied according to an identical protocol and light-cured for 20 seconds.

### Subgroup C (Bioactive Sealant):

Bioactive calcium-phosphate-releasing sealant (Biocoat, Premier Dental, Plymouth Meeting, PA, USA) was dispensed using manufacturer-provided applicator tips, manipulated to ensure complete coverage, and photopolymerized for 20 seconds.

### Artificial Aging Protocol:

All specimens underwent thermocycling simulation (Thermocycler 1100, SD Mechatronik, Feldkirchen-Westerham, Germany) comprising 10,000 cycles alternating between water baths maintained at 5±2°C and 55±2°C, with 30-second dwell times and 5-second transfer intervals.^14^

### Microleakage Assessment:

Following thermocycling, specimens designated for microleakage evaluation (n=5 per subgroup) received a two-layer acid-resistant nail varnish coating, with 1 mm margins around sealed fissures. Specimens were immersed in a 1% aqueous solution of methylene blue (pH 7.0) at 37°C for 48 hours to facilitate dye penetration into deficient marginal seal sites. Post-immersion, teeth were rinsed and air-dried before longitudinal buccolingual sectioning using a water-cooled slow-speed diamond disc (Isomet 1000, Buehler, Lake Bluff, IL, USA), yielding four slices per specimen (mesial, two central, distal sections). Sectioned specimens were examined under stereomicroscopy (SWIFT M3600D, SWIFT Instruments Inc., Boston, MA, USA) at ×40 magnification by two calibrated examiners (inter-examiner reliability: κ=0.87), blinded to group allocation.^2^

### Microtensile Bond Strength Testing:

Sixty sealed molars (10 specimens per subgroup) were allocated for microtensile bond strength (μTBS) evaluation. Each tooth was longitudinally sectioned perpendicular to the enamel-sealant interface using a water-cooled precision diamond saw to fabricate rectangular beam specimens with standardized dimensions: 1.0 mm thickness, 7.0 mm length, and 1.0 mm width, verified using a digital Vernier caliper (Mitutoyo Corporation, Kawasaki, Japan; accuracy ±0.01 mm). Only beams with intact interfaces and uniform cross-sectional areas were included in testing. Individual beams were secured to a universal testing machine (Model MEM 2000, EMIC Equipment and Systems Ltd., São José dos Pinhais, Brazil) using cyanoacrylate adhesive (Super Bonder, Henkel Loctite, São Paulo, Brazil) to fix specimen ends to custom metal jigs, ensuring precise alignment of the enamel-sealant interface perpendicular to the loading axis. Tensile force was applied at a constant crosshead speed of 1.0 mm/minute until catastrophic bond failure, identified by an audible crack and a sudden load drop. Maximum failure load (Newtons) was recorded automatically, and μTBS values were calculated using the formula:

μTBS (MPa) = Maximum load (N) / Cross-sectional area (mm^2^).

### Failure mode analysis:

Immediately following bond strength testing, fractured surfaces of all debonded specimens were examined under stereomicroscopy (SWIFT M3600D,×40 magnification) by two independent calibrated observers (inter-rater reliability: κ=0.91) masked to experimental groups. Failure patterns were classified as adhesive, cohesive, and admixed.^15^

### Statistical analysis:

The collected data were analyzed using SPSS software (version 25.0; IBM Corp., Chicago, IL, USA). One-way analysis of variance (ANOVA) was conducted, followed by Tukey’s post hoc test for multiple comparisons. p < 0.05.

## RESULTS

### Microleakage scores:

Table-I displays the microleakage of different PFS bonded to enamel conditioned using different techniques. Maximum marginal leakage was observed in Group-2A (ECL + Conventional PFS) (30.54 ± 0.42). In contrast, minimum leakage was witnessed in Group-1C (PA + Bioactive PFS) (12.88 ± 0.32). Intergroup comparison analysis indicated that Group-1A (PA + Conventional PFS) (29.11 ± 0.21) and Group-2A (ECL + Conventional PFS) presented no significant difference in marginal leakage score. Likewise, Group-1B (PA + Fluoride-releasing PFS) (12.94 ± 0.22), Group-1C (PA + Bioactive PFS) (12.88 ± 0.32), Group-2B (ECL + Fluoride-releasing PFS) (13.12 ± 0.32), and Group-2C (ECL + Bioactive PFS) (13.01 ± 0.24) also demonstrated no significant disparity in the microleakage scores (p > 0.05).

**Table-I:** Microleakage of different pits and fissure sealant bonded to enamel conditioned using ECL and PA.

Tested groups	Mean ± SD (µm)	p-value*
Group-1A: PA + Conventional PFS	29.11 ± 0.21^a^	< 0.05
Group-2A: ECL + Conventional PFS	30.54 ± 0.42^a^	
Group-1B: PA + Fluoride-releasing PFS	12.94 ± 0.22^b^	
Group-2B: ECL + Fluoride-releasing PFS	13.12 ± 0.32^b^	
Group-1C: PA + Bioactive PFS	12.88 ± 0.32^b^	
Group-2C: ECL + Bioactive PFS	13.01 ± 0.24^b^	

* ANOVA; Pits and fissure sealant (PFS), Phosphoric acid (PA), Er, Cr: YSGG laser (ECL). Different superscript lowercase letters indicate a statistically significant difference (p < 0.05).

### μTBS assessment:

Table-II exhibits the μTBS of different PFS bonded to enamel conditioned using different techniques. Maximum bond strength was observed in Group-1C (PA + Bioactive PFS) (11.52 ± 0.43 MPa). While minimum bond strength integrity was displayed by Group-2A (ECL + Conventional PFS) (7.01 ± 0.75 MPa). Intergroup comparison revealed that Group-1A (PA + Conventional PFS) (7.22 ± 0.54 MPa) and Group-2A (ECL + Conventional PFS) presented no significant difference in bond strength. (p > 0.05) Likewise, Group-1B (PA + Fluoride-releasing PFS) (11.48 ± 0.37 MPa), Group-1C (PA + Bioactive PFS) (11.52 ± 0.43 MPa), Group-2B: ECL + Fluoride-releasing PFS (11.21 ± 0.32 MPa), and Group-2C (ECL *+* Bioactive PFS) (11.31 ± 0.30 MPa) also demonstrated no significant disparity in the μTBS scores. (p > 0.05).

**Table-II:** µtbs of different pits and fissure sealant bonded to enamel conditioned with Er, Cr:YSGG, and PA.

Tested groups	Mean ± SD (MPa)	p-value*
Group-1A: PA + Conventional PFS	7.22 ± 0.54^a^	***< 0.05***
Group-2A: ECL + Conventional PFS	7.01 ± 0.75^a^	
Group-1B: PA + Fluoride-releasing PFS	11.48 ± 0.37^b^	
Group-2B: ECL + Fluoride-releasing PFS	11.21 ± 0.32^b^	
Group-1C: PA + Bioactive PFS	11.52 ± 0.43^b^	
Group-2C: ECL + Bioactive PFS	11.31 ± 0.30^b^	

* ANOVA; Pits and fissure sealant (PFS), Phosphoric acid (PA), Er, Cr: YSGG laser (ECL).Different superscript lowercase letters indicate a statistically significant difference (p < 0.05).

### Failure mode analysis:

**Fig.1:**
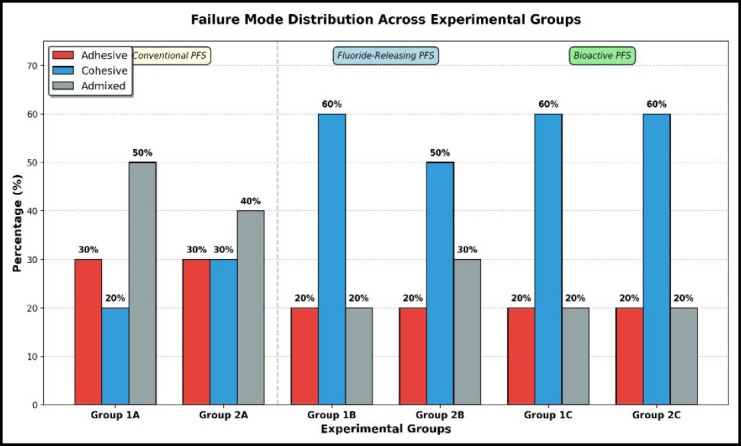
This grouped bar chart displays the three failure modes (Adhesive, Cohesive, Admixed) for each experimental group. The chart allows direct comparison of failure patterns between Group-1 (37% phosphoric acid) and Group-2 (Er, Cr: YSGG laser) across different sealant types (A: Conventional, B: Fluoride-releasing, C: Bioactive).

Fig. 1 shows the failure mode analysis across the tested groups. Groups 1B, 2B, 1C, and 2C had the most cohesive failures. Whereas Groups 1A and 2A predominantly exhibited admixed failures.

## DISCUSSION

The present investigation evaluated two null hypotheses regarding enamel conditioning methods and pit and PFS performance in primary dentition. The first hypothesis, proposing equivalent microleakage and μTBS between Er: YSGG laser and phosphoric acid conditioning within identical sealant groups, was accepted based on statistical equivalence across all parameters. Conversely, the second hypothesis was rejected, as fluoride-releasing and bioactive sealants demonstrated significantly superior μTBS values and reduced marginal leakage compared to conventional formulations.

Marginal adaptation constitutes the primary determinant of sealant longevity, as optimal interfacial seal integrity prevents bacterial microleakage and subsequent caries development.^16^ The inverse relationship observed between microleakage scores and bond strength corroborates findings by AlQahtani et al., reflecting fundamental adhesive mechanics wherein superior interfacial bonding produces continuous, defect-free seals that resist fluid penetration.^17^ This phenomenon validates dye penetration methodology as a reliable surrogate marker for clinical seal integrity.

In the present study, bioactive sealants demonstrated enhanced performance through synergistic mechanisms that combined chemical and mechanical retention. Functional ion release (calcium, phosphate, fluoride) establishes chemical interactions with enamel hydroxyapatite, supplementing conventional micromechanical interlocking.^18^ Progressive mineralization at the enamel-sealant interface reinforces marginal seal integrity over time, explaining why bioactive materials demonstrate improved performance following artificial aging while conventional sealants undergo hydrolytic degradation.^12^ The hydrophilic nature of bioactive formulations facilitates superior wetting of etched enamel, particularly advantageous in moisture-contaminated clinical scenarios where hydrophobic conventional resins exhibit compromised penetration.^17^ Similarly, fluoride-releasing sealants (Clinpro) demonstrated comparable performance attributable to their unfilled resin composition, conferring exceptionally low viscosity.^2^ Surface science principles dictate that effective liquid spreading requires substrate surface energy exceeding liquid surface tension; low-viscosity sealants achieve superior fissure penetration, and elevated bond strengths consistent with findings by Rirattanapong et al.^19^

The statistical equivalence between ECL and PA conditioning validates laser irradiation as a viable alternative when optimized parameters are employed. Conventional 37% phosphoric acid generates uniform Type I/II etching patterns, facilitating extensive micromechanical retention. Er, Cr: YSGG laser (2780 nm wavelength) achieves comparable surface morphology through hydrokinetic ablation—rapid water vaporization inducing microexplosions that ablate enamel while creating retentive topography without chemical exposure.^20^,^21^ Labunet et al.’s systematic review identified multiple studies demonstrating equivalent laser-acid outcomes when appropriate power settings (2.0–3.5 W) were used.^22^ The present study’s optimized protocol (3.5 W, 140 μs pulse duration, 20 Hz frequency, 15-second exposure with air-water cooling) successfully replicated acid-etching microretention, contrasting with studies by Hoshing et al. employing suboptimal settings that produced inferior bonding.^23^

Failure mode analysis revealed that cohesive failures predominated in the fluoride-releasing and bioactive groups, indicating that interfacial bond strength exceeded material cohesive strength—the optimal clinical outcome, validating superior performance metrics.^24^ Conventional sealants exhibited admixed failures combining adhesive and cohesive components, suggesting heterogeneous bonding quality wherein material properties rather than conditioning technique constituted the performance-limiting factor. This underscores that sealant formulation and surface preparation synergistically determine success, with advanced materials maximizing bonding potential from either conditioning method.

### Limitations:

This investigation is inherently limited by its in vitro design, which, despite simulating thermocycling, cannot fully replicate the complex biochemical, mechanical, and microbiological dynamics of the oral environment. The small subgroup sample sizes allocated to microleakage evaluation (n=5) limit the statistical power of these comparisons. Furthermore, the exclusive use of primary mandibular molars limits the generalizability of the findings to the broader primary dentition and permanent teeth.

### Study novelty:

This study represents the first comparison of three types of sealants—conventional, fluoride-releasing, and bioactive—employing two distinct conditioning methods. It marks the inaugural application of the Er, Cr: YSGG laser for bonding pit-and-fissure sealants in primary teeth. Additionally, it concurrently evaluates two critical parameters: microleakage, a predictor of clinical failure, and µTBS, an indicator of mechanical integrity.

### Contributions to Dental Literature:

This study provides the first comprehensive comparison of bioactive, fluoride-releasing, and conventional pit-and-fissure sealants in primary teeth. The findings suggest that bioactive sealants perform better. The interaction between the material and the conditioning method is crucial: conventional sealants necessitate acid etching, whereas advanced materials are compatible with laser conditioning. Clinicians managing high-caries-risk pediatric patients should consider bioactive or fluoride-releasing sealants as the preferred formulations, irrespective of the conditioning modality employed.

## CONCLUSION

Fluoride-releasing and bioactive pit-and-fissure sealants demonstrate significantly superior performance compared to conventional formulations. Er, Cr: YSGG laser conditioning produces outcomes equivalent to phosphoric acid etching when optimized parameters are used, establishing laser irradiation as a viable pediatric alternative. These findings support the adoption of advanced sealant materials to maximize preventive efficacy, though clinical validation remains warranted.

### Future recommendations:

Comparative studies evaluating emerging enamel conditioning modalities—such as non-thermal plasma and Er: YAG laser—alongside a broader portfolio of next-generation bioactive sealant materials are warranted to optimize preventive protocols in pediatric dentistry.
